# NO gas sensing at room temperature using single titanium oxide nanodot sensors created by atomic force microscopy nanolithography

**DOI:** 10.3762/bjnano.7.97

**Published:** 2016-07-20

**Authors:** Li-Yang Hong, Heh-Nan Lin

**Affiliations:** 1 Department of Materials Science and Engineering, National Tsing Hua University, Hsinchu 30013, Taiwan

**Keywords:** atomic force microscopy nanolithography, photo-activation, photo-recovery, resistive NO gas sensor, titanium oxide nanodot sensor

## Abstract

In this work, the fabrication of single titanium oxide nanodot (ND) resistive sensors for NO gas sensing at room temperature is reported. Two atomic force microscopy nanolithography methods, nanomachining and nano-oxidation, are employed. A single titanium nanowire (NW) is created first along with contact electrodes and a single titanium oxide ND is subsequently produced in the NW. Gas sensing is realized by the photo-activation and the photo-recovery approaches. It is found that a sensor with a smaller ND has better performance than a larger one. A response of 31%, a response time of 91 s, and a recovery time of 184 s have been achieved at a concentration of 10 ppm for a ND with a size of around 80 nm. The present work demonstrates the potential application of single metal oxide NDs for gas sensing with a performance that is comparable with that of metal oxide nanowire gas sensors.

## Introduction

In recent years, gas sensors have been widely used in a variety of fields, such as medical diagnosis [1–2], environmental monitoring [3] and combustion emission control [4]. Among all types of gas sensors, resistor-type gas sensors based on semiconducting metal oxide nanomaterials [5–8] are more attractive than conventional devices [9]. The advantages include high sensitivity, high stability, fast detection and recovery, low power consumption, relatively low cost, and small size [9–10]. These advantages enable semiconducting metal oxide sensors to be implemented on integrated circuits for portable applications [5,11].

Semiconducting metal oxide gas sensors generally need to work at high temperatures due to the high energy required for surface reactions [5,12–13]. However, a high operating temperature results in issues with durability and reliability of the device [5]. To overcome this drawback, light-assisted approaches including photo-activation [12–21] and photo-recovery [22–23] have been shown effective to enable gas sensing at room temperature.

The sensing material in a semiconducting metal oxide sensor is commonly synthesized by a bottom-up approach, such as chemical vapour deposition [11,15–16,23], thermal deposition [12], solution growth [19], and electrospinning [24]. Alternatively, a lithographic approach is also suitable for producing the sensing metal oxide material. Previously, atomic force microscopy (AFM) nano-oxidation has been utilized for the fabrication of titanium oxide nanowire (NW) gas sensors [25–26].

NO gas sensing at low concentrations is beneficial for human health [1–2] and environmental monitoring [3]. Various types of metal oxide nanomaterials have been utilized for NO or NO_2_ gas sensing, e.g., SnO_2_ [12,15–17], ZnO [13–14,17–20,23], In_2_O_3_ [22], and TiO_2_ [24,27–29]. AFM nanolithography is a valuable technique for the fabrication of nanostructures and sensors [30–31]. Recently, we have reported on the fabrication of single titanium oxide nanodot (ND) ultraviolet (UV) sensors by AFM nanomachining and nano-oxidation [32]. In the present work, the application of single titanium oxide ND sensors for NO gas sensing at room temperature is reported. The performance of the ND gas sensors compares reasonably with metal oxide NW gas sensors reported in the literature.

## Experimental

**Figure 1:**
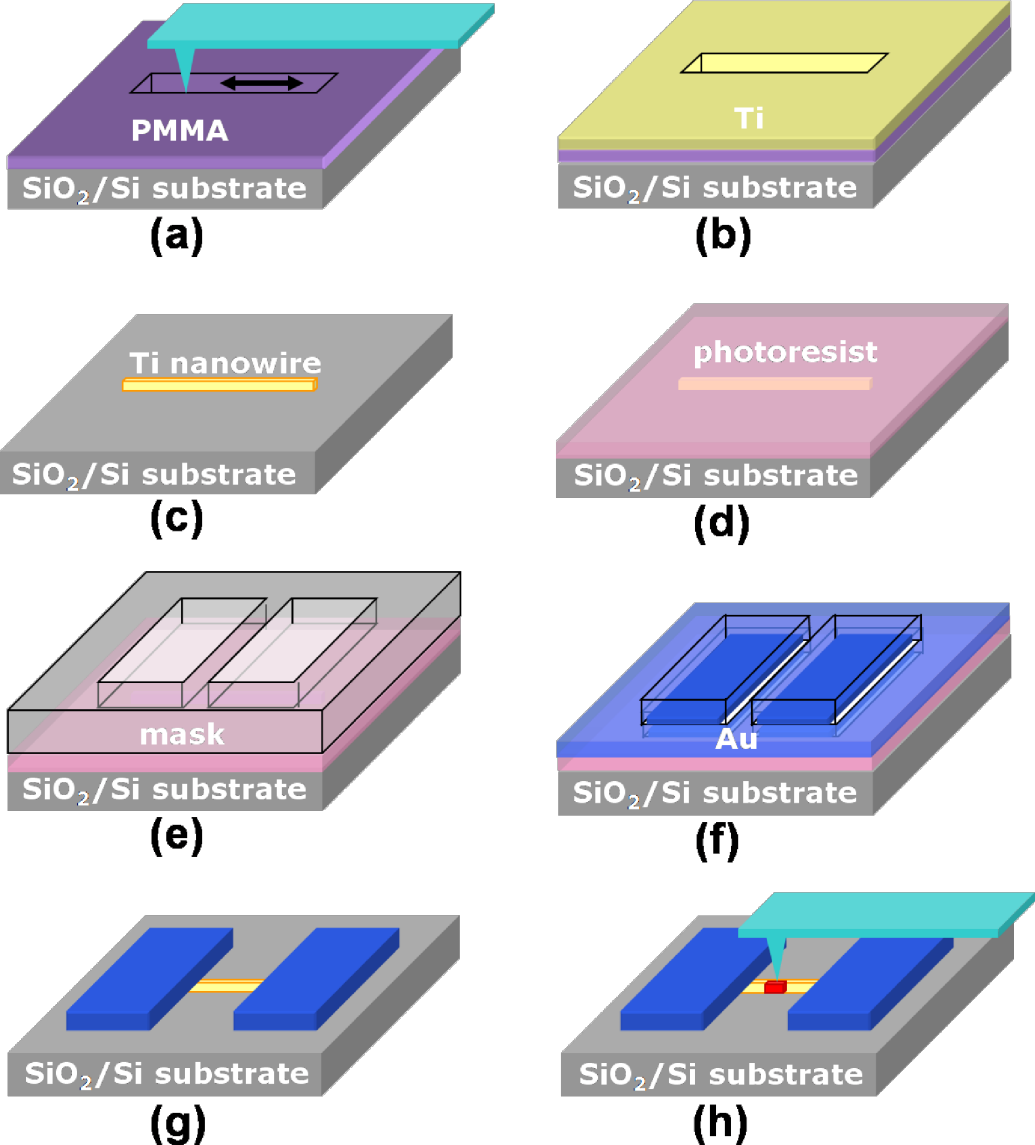
A schematic diagram showing the fabrication of a single titanium oxide ND gas sensor. (a) PMMA spin-coated and AFM nanomachining, (b) Ti deposition, (c) PMMA lift-off, (d) photoresist spin-coated, (e) exposure and development, (f) Au deposition, (g) photoresist lift-off, and (h) AFM nano-oxidation. (Adapted from [32]).

A schematic diagram of the AFM nanolithography [30,32] procedure is shown in Figure 1. A 40 nm thick poly(methylmethacrylate) (PMMA) film was spin-coated on a Si substrate that had a thick oxide layer. By using an AFM (Smena, NT-MDT, Russia), a straight nanogroove was generated in the PMMA film. A Ti film was deposited by electron-beam deposition and a single Ti NW was created after lift-off. Au contact electrodes were subsequently created on the sides of the NW by standard photolithography. The details can be found in our previous reports [33–34]. For nano-oxidation, the tip was moved to the middle of the NW and a voltage pulse (−10 V and 500 ms) was applied to the tip. A single titanium oxide ND sensor was thus obtained [32]. The morphologies of the NW and the ND were examined by another AFM (Dimension Icon, Bruker, U.S.A.).

The fabricated ND sensor was put in a vacuum chamber and connected to a Keithley 2400 source measure unit. Prior to a sensing cycle, the chamber was pumped to low vacuum (ca. 10^−3^ Torr) and the valve was closed. A mixture of NO (500 ppm) and N_2_ gas was injected into the chamber and a sensing cycle was started. With a mass flow controller, a specific NO concentration was quickly established in the chamber for sensing. After a sufficient sensing time (which is shown in blue in the current-response figures), the chamber was evacuated again for NO desorption (which is shown in white in the current-response figures). After the pumping, the sensing cycle ended and the next cycle was started. The time-dependent current of the sensor at a bias voltage of 10 or 5 V was recorded. A UV light-emitting diode (310 nm at 0.3 mW·cm^−2^) was placed above the sensor for UV-assisted sensing. Two sensing modes were applied. For the first, called UV-activation mode, the sensor was under constant UV illumination through the whole measurement. For the second, called UV-recovery mode, the UV light was turned on after sensing for NO desorption. (Note that the chamber was under pumping.) The UV light was then turned off after a certain time. (Note that the chamber was still under pumping after the light was turned off.)

## Results and Discussion

**Figure 2:**
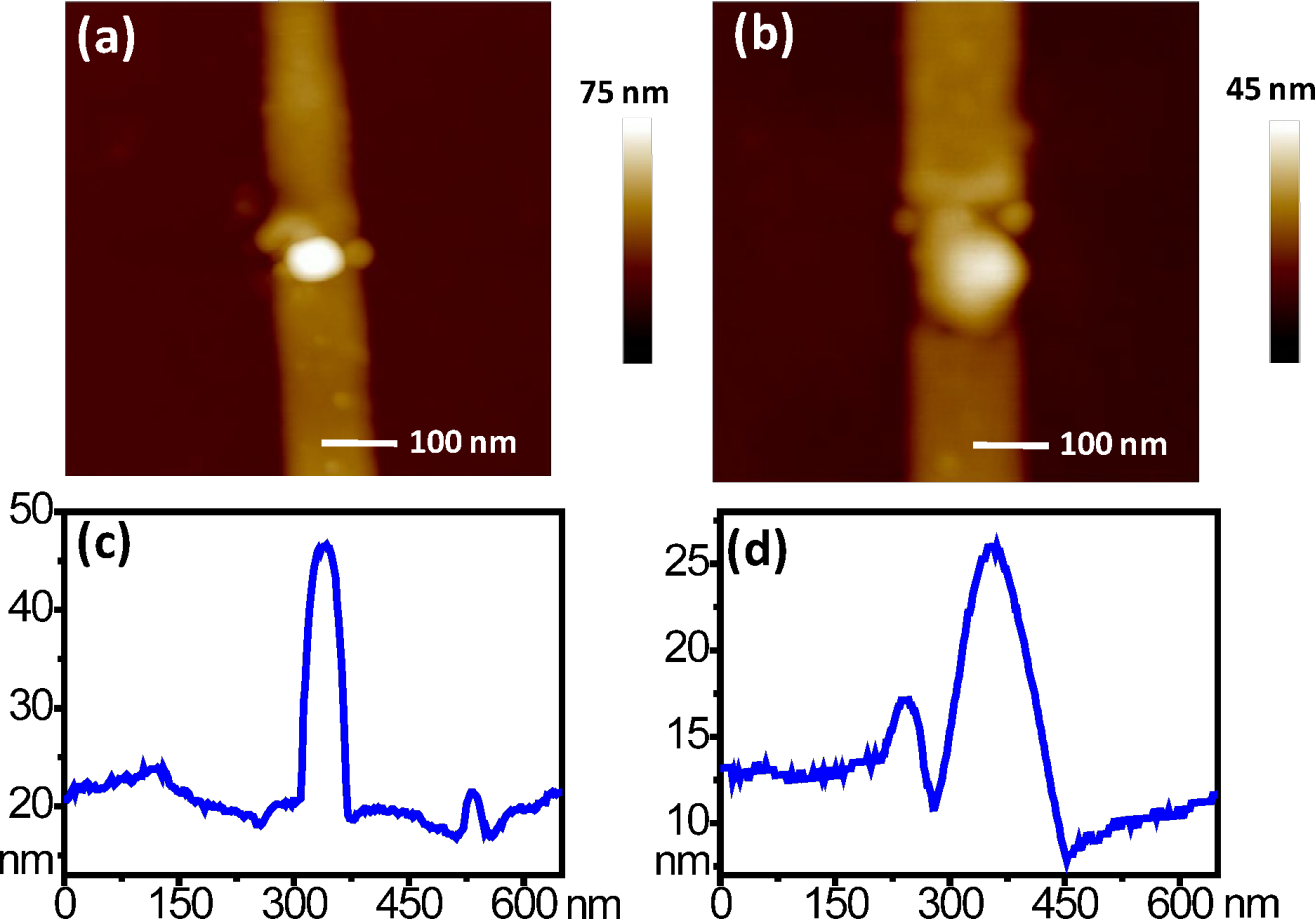
(a,b) AFM topographic images of the two NDs of sensors A and B, respectively. (c,d) Cross-sectional plots of the two NDs.

Two ND gas sensors with different sizes have been fabricated. The smaller one will be called sensor A and the larger one sensor B. An AFM topographic image of sensor A is shown in Figure 2a and a cross-sectional plot is shown Figure 2c. The ND has a length, a width and a height of around 50, 80 and 26 nm, respectively. A topographic image of sensor B is shown in Figure 2b and a cross-sectional plot is shown in Figure 2d. The ND is larger with a length, a width and a height of around 134, 120 and 16 nm, respectively. The surface to volume ratio is an important factor for the sensing performance. Taking the NDs as simple rectangular cuboids, the estimated volume and surface area (only five surfaces) for sensor A are around 104 × 10^3^ nm^3^ and 10.8 × 10^3^ nm^2^, respectively. The surface to volume ratio is roughly 0.103 nm^−1^. The estimated volume and surface area for sensor B are around 257 × 10^3^ nm^3^ and 24.2 × 10^3^ nm^2^, respectively. The surface to volume ratio is roughly 0.094 nm^−1^. Therefore, sensor A has a larger surface to volume ratio.

The generation of the NDs after nano-oxidation shown in Figure 2 is clear evidence of the formation of titanium oxide. Also, Auger electron spectroscopy analysis confirmed that the Ti was oxidized [32]. The compositions of the NDs, however, cannot be exactly determined and are simply TiO*_x_*. Also, sensor A has a larger resistance due to the smaller ND size. (The current–voltage relationships of the two ND sensors before NO sensing are shown in Figure S1 in Supporting Information File 1.) We discussed the electrical properties of the sensors in our previous report [32]. A TiO*_x_* ND behaves like an n-type semiconductor due to oxygen vacancies. When NO molecules (or O_2_ molecules under ambient environment) adsorb on the ND surface, they become negatively charged by catching electrons. The conductance of the ND with NO (or O_2_) adsorption is thus smaller than that in the pristine state of the ND (i.e., without adsorbed molecules). Under UV illumination, the ND conductance becomes larger since the adsorbed NO (or O_2_) molecules are neutralized by photo-generated holes and leave the ND surface [35]. Detailed mechanisms will be discussed later.

**Figure 3:**
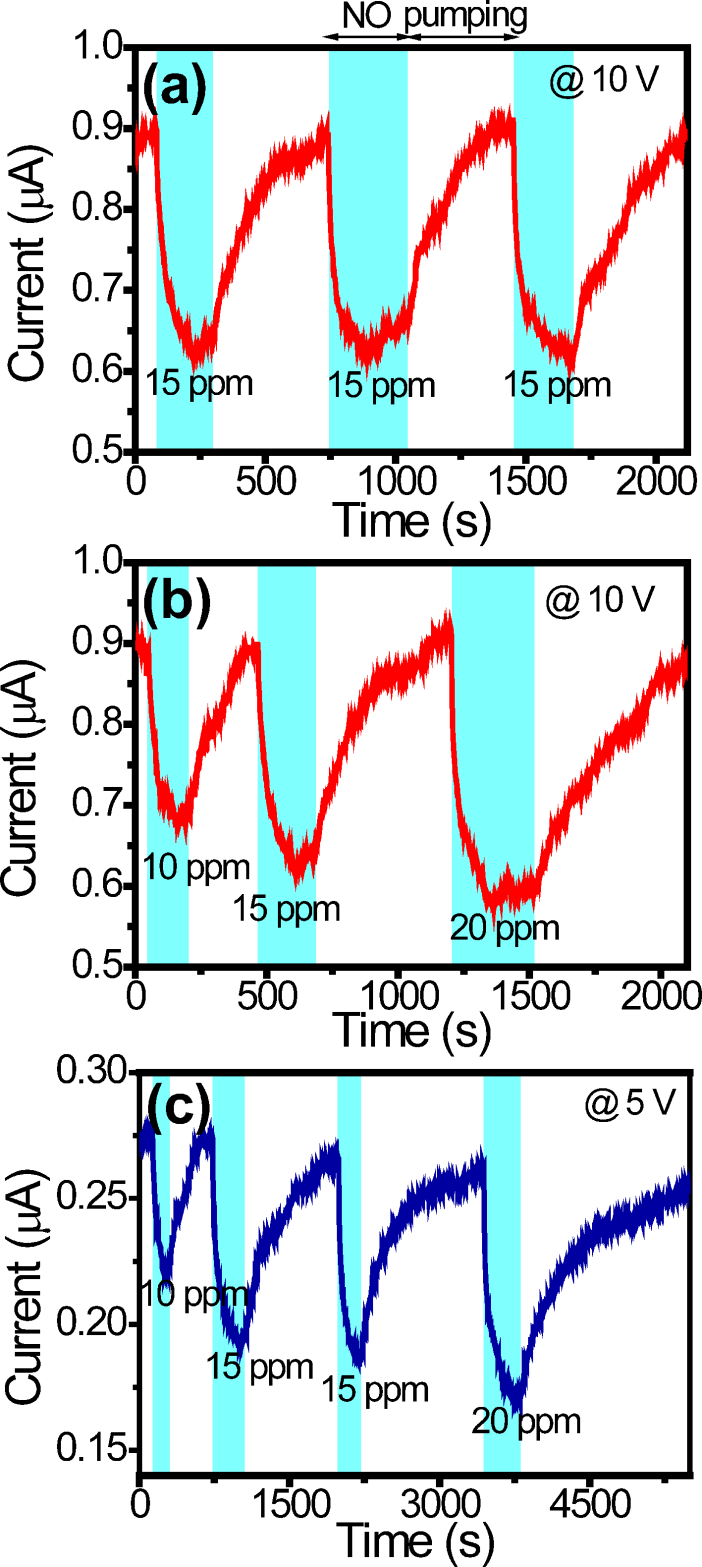
(a) The current response of sensor A at 10 V and 15 ppm NO, and (b,c) the current responses at 10 and 5 V, respectively, for various concentrations in the UV-activation mode. The blue regions indicate NO presence and the white regions indicate pumping.

Figure 3a shows the current response of sensor A at 15 ppm NO and a bias of 10 V in the UV-activation mode. (Note that it was confirmed first that pure N_2_ gas had no effect on the sensor current.) The starting current (ca. 0.9 µA) is larger than the current under ambient environment since the sensor is under UV irradiation. The current decreases after NO injection (blue region) and stabilizes after a certain time. (The NO exposure times in the blue regions in Figure 3a are around 215, 305 and 230 s, respectively.) It gradually increases when the NO gas is pumped out (white region). As can be seen, the current variations are consistent for the three cycles. Figure 3b shows the current response at 10, 15 and 20 ppm. (The exposure times in Figure 3b are 155, 230 and 315 s, respectively.) It is clear that the current decrease becomes larger as the concentration rises, because more molecules adsorb on the surface. Furthermore, it takes longer time for the current to return to its original value as the concentration gets higher. Figure 3c shows the current response at 10, 15, and 20 ppm NO and at 5 V. (The exposure times in Figure 3c are 170, 315, 230 and 370 s.) In comparison with Figure 3b, it takes longer time for the current to return to its original value. The faster recovery (i.e., faster NO desorption) in Figure 3b is caused by the self-heating effect [36–37]. When operating at a larger bias, the sensor temperature is higher owing to more Joule heat. The rate of desorption increases accordingly. A larger operating voltage is thus beneficial for sensing, but at the cost of higher risk of breaking down.

**Figure 4:**
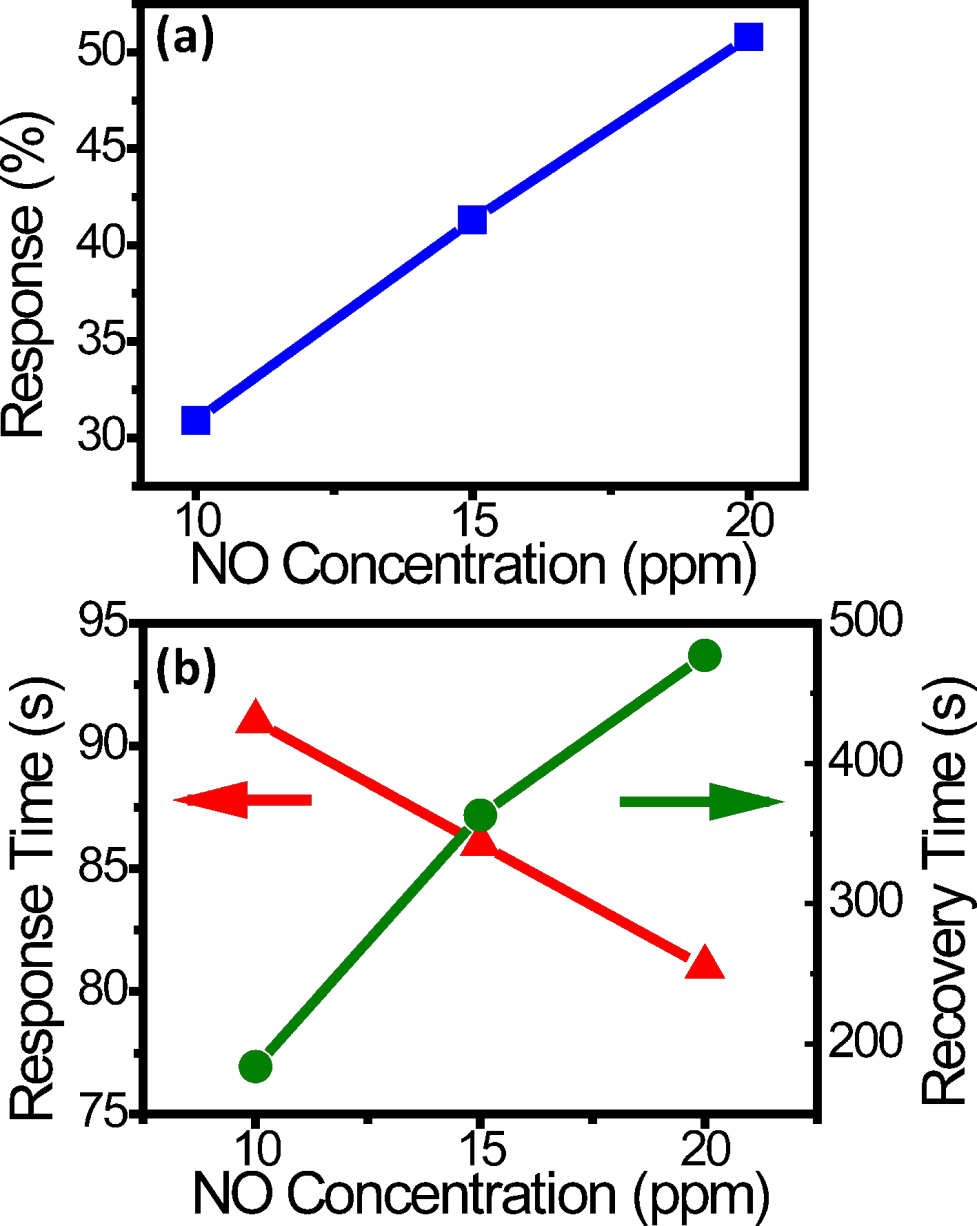
(a) Response and (b) response time and recovery time as a function of the concentration for sensor A at 10 V in the UV-activation mode calculated from Figure 3b.

Figure 4 shows the performance of sensor A at 10 V calculated from Figure 3b. The response *S* is defined as *S* = Δ*R*/*R*_0_, where Δ*R* is the resistance increase after NO adsorption and *R*_0_ the initial resistance in a blue region in Figure 3b. The responses are 31, 41, and 52% for 10, 15, and 20 ppm, respectively, and plotted in Figure 4a. (The resistances before and after NO adsorption are shown in Table S1 in Supporting Information File 1.) The response time *t*_res_ is defined as the time required for the current to decrease to 10% of the initial current during NO exposure. The results are 91, 86, and 81 s for 10, 15, and 20 ppm, respectively, and plotted in Figure 4b. The recovery time *t*_rec_ is defined as the time required for the current to increase to 90% of the original current when NO is being pumped out. The results are 184, 363, and 477 s for 10, 15, and 20 ppm, respectively, and also plotted in Figure 4b. Response and recovery time both increase as the concentration rises. Since there are more adsorbed molecules on the ND surface at a higher concentration, it is reasonable that the response becomes larger. Also, it takes a longer time for the molecules to leave the surface during recovery. On the other hand, the response time has an opposite trend. This can be attributed to faster molecular adsorption on the ND surface at a higher concentration.

**Figure 5:**
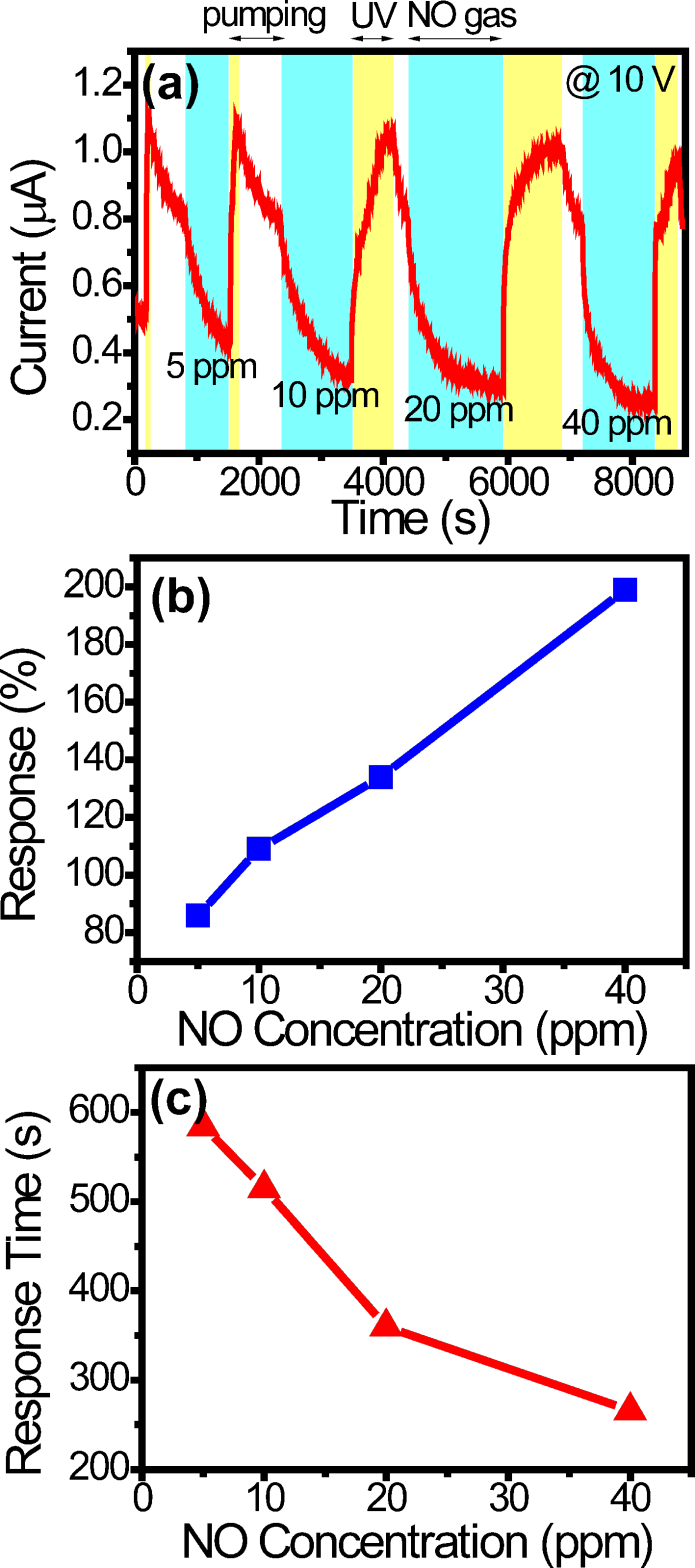
(a) The current response of sensor A at 10 V in the UV-recovery mode. (b) Response and (c) response time as a function of the concentration.

Figure 5a shows the current response of sensor A at 10 V in the UV-recovery mode. At the beginning, the UV irradiation is turned on for O_2_ desorption (the chamber is also under pumping) and the current rises immediately. When the current reaches a high value (roughly 1.1 µA in Figure 5a), the UV irradiation is then turned off. The current gradually decreases due to re-adsorption of O_2_. (Note that re-adsorption occurs due to the low vacuum in spite that the chamber is still under pumping.) When it reaches an approximately steady value of 0.8 µA, NO is injected and the sensing starts. (A finer time scale current response is shown in Figure S2 in Supporting Information File 1 and it reveals the current is approximately steady at 0.8 µA.) After a sufficient sensing time, the UV irradiation is turned on (yellow region) for NO desorption and the process repeats. From the current response, the responses are 86, 108, 134, and 199% for 5, 10, 20, and 40 ppm, respectively, and plotted in Figure 5b. The response times are 584, 515, 360, and 266 s, respectively, and plotted in Figure 5c. It is difficult to determine the recovery times in the UV-recovery mode and, therefore, they are not shown.

The responses in Figure 5b are roughly three times as high as those in Figure 4a, suggesting that the UV-recovery mode is more sensitive. In the UV-activation mode, desorption occurs to some extent during NO exposure because of the UV irradiation. On the contrary, in the UV-recovery mode, there is no desorption during NO exposure. Consequently, there are more adsorbed molecules and the response is larger. The response times in Figure 5c are much longer than those in Figure 4b, suggesting that the UV-recovery mode has a slower response. This can be explained again by molecular adsorption and desorption. In the UV-activation mode, the adsorption and desorption of NO molecules rapidly reach a dynamic equilibrium under UV illumination during NO exposure. In the UV-recovery mode, it takes much longer time to reach full adsorption during NO exposure. Therefore, the response is much slower.

**Figure 6:**
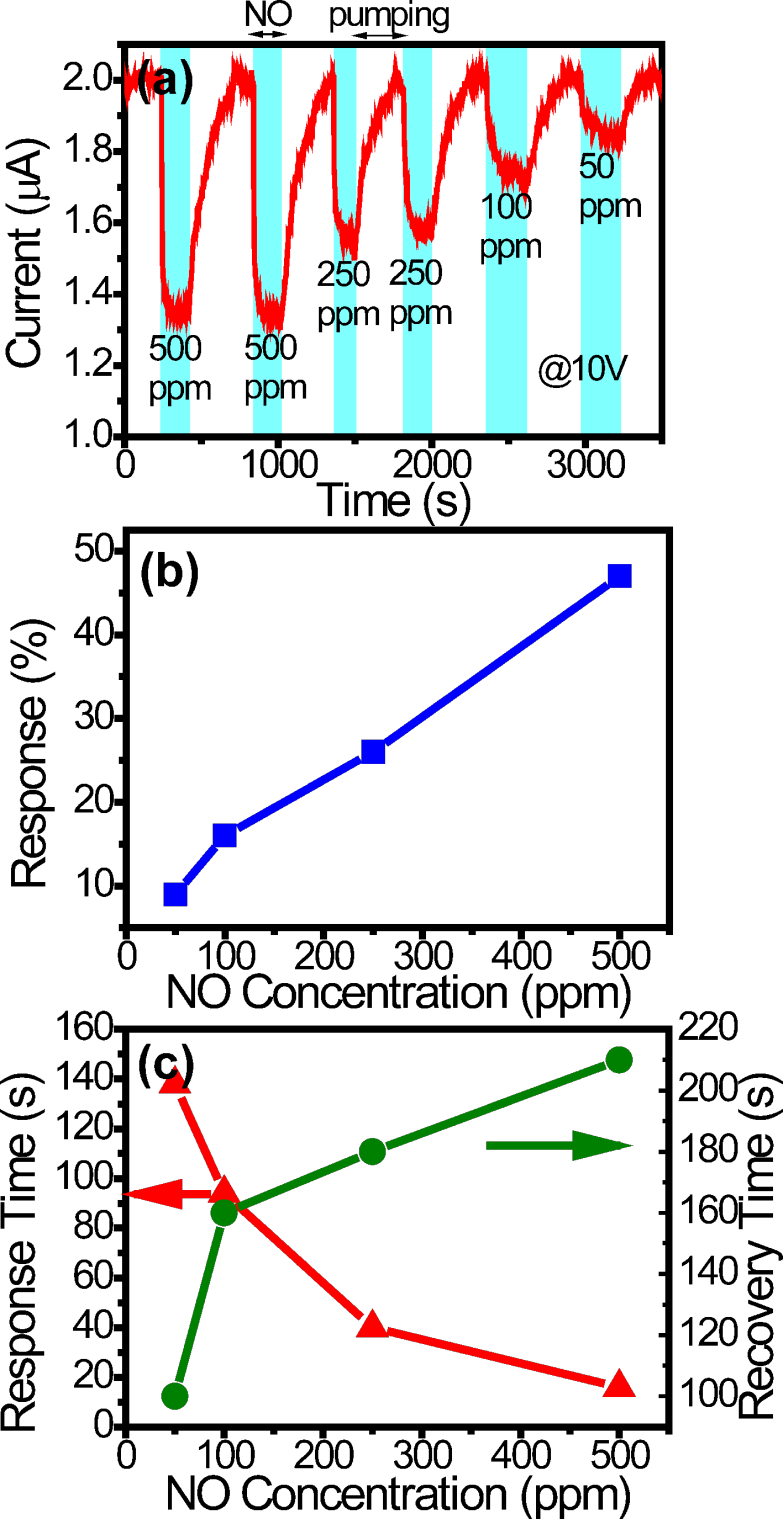
(a) The current responses of sensor B at 10 V in the UV-activation mode. (b) The response and (c) the response time and the recovery time versus the concentration.

Figure 6a shows the current response of sensor B at 10 V between 50 and 500 ppm in the UV-activation mode. (The current response at 5 V in the UV-activation mode is shown in Figure S3 in Supporting Information File 1 for comparison.) The responses are 9, 16, 27, and 47% for 50, 100, 250, and 500 ppm, respectively, and plotted in Figure 6b. (The resistances before and after NO adsorption are also shown in Table S1 in Supporting Information File 1.) As expected, the response increases as the NO concentration rises. The response times are 138, 94, 40, and 16 s, and the recovery times are 100, 160, 180, and 210 s for the four concentrations, respectively. They are all plotted in Figure 6c. Similarly to the results in Figure 4, the response time decreases and the recovery time increases as the concentration rises.

A summary of the responses, the response times, and the recovery times of the two sensors obtained at 10 V in the UV-activation mode is shown in Table 1. As has been discussed previously, the response and the recovery time both increase as the concentration rises, whereas the response time has an opposite trend. Furthermore, sensor A has larger and faster response, but longer recovery than sensor B. The first two benefits can be explained by two factors. One is that the ND in sensor A has a larger surface to volume ratio. The other is due to the fact that the depletion layer thickness after adsorption is roughly equal to the Debye length [5,17,38]. Therefore, the conduction channel in the ND of sensor A after adsorption is narrower than that in sensor B. The combination of the two factors makes the smaller sensor A more sensitive. The longer recovery of sensor A may be explained by the following argument: There are more photo-generated charge carriers in sensor B than in sensor A under UV illumination since sensor B is larger. The adsorbed NO molecules can thus be more efficiently neutralized and leave the ND surface. As a result, sensor B has shorter recovery.

**Table 1:** A summary of the responses, the response times, and the recovery times for the two sensors obtained at 10 V in the UV-activation mode. *C* is the NO concentration.

sensor	*C* (ppm)	*S* (%)	*t*_res_ (s)	*t*_rec_ (s)

A	10	31	91	184
	15	41	86	363
	20	52	81	477
B	50	9	138	100
	100	16	94	160
	250	27	40	180
	500	47	16	210

Additionally, it is worth mentioning that the background N_2_ pressure was 2000 times that of the NO pressure and roughly a few percents of one atmosphere in the above measurements. In order to verify if a higher N_2_ pressure affected the sensing results, a measurement was conducted with the N_2_ pressure raised to one atmosphere after NO sensing. It was found that there was no change in the current response. (The current response is shown in Figure S4 in Supporting Information File 1.) It is therefore reasonable to expect that the present sensing results will not change in a high-pressure N_2_ environment.

**Figure 7:**
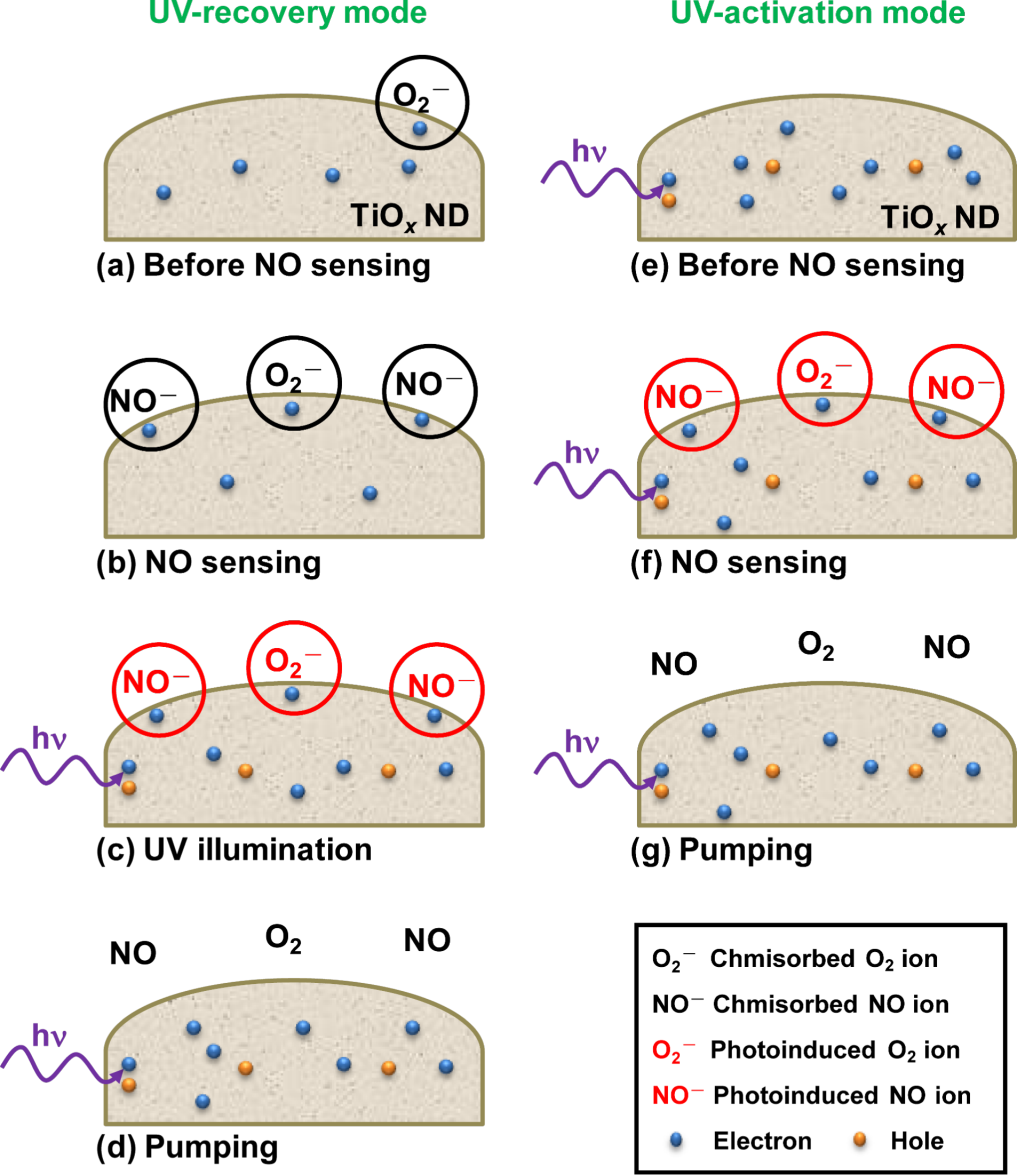
Diagram illustrating NO gas sensing mechanisms for the single titanium oxide ND sensor in the UV-recovery and the UV-activation modes.

Possible mechanisms for NO or NO_2_ sensing by using semiconducting metal oxide nanomaterials have been proposed and discussed in previous works [19–20,26,38]. Based on these works, Figure 7 shows the gas sensing mechanisms for the single titanium oxide ND sensor in the UV-recovery and the UV-activation modes. In the UV-recovery mode, O_2_ and NO molecules react with electrons to form chemisorbed oxygen ions (O_2_^−^_(ads)_) and nitric oxide ions (NO^−^_(ads)_) prior to UV irradiation, which are shown in Figure 7a and Figure 7b. The chemical reactions for NO are described by [20,38]:

[1]
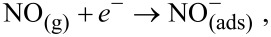


[2]
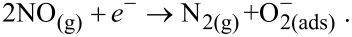


Without UV illumination, the chemisorbed ions are too stable to be removed by pumping. Under UV illumination, the ions are first neutralized by photogenerated holes and then react again with photogenerated electrons [13], which is shown in Figure 7c. The chemical reactions for NO can be similarly described by:

[3]
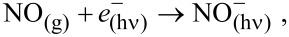


[4]
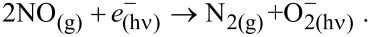


Unlike the chemisorbed ions, the photoinduced ions (O_2_^−^_(hν)_ and NO^−^_(hν)_) are only weakly bound to the ND surface. They can be easily removed by pumping as shown in Figure 7d. In the UV-activation mode, the ND is under continuous UV irradiation. There are no chemisorbed ions before NO sensing, which is shown in Figure 7e. Photoinduced ions are then created after NO injection as shown in Figure 7f. The photoinduced ions are finally removed by pumping as shown in Figure 7g.

Table 2 shows a comparison of sensing performances of NO or NO_2_ metal oxide sensors operated in the photo-activation mode at room temperature reported in the literature. Although not better, the performance of sensor A compares reasonably with these reported results. Furthermore, it can be seen that the performances of metal oxides with Au are much better those of pure metal oxides, which is due to the plasmonic effect [18,20]. It is expected that the present ND sensors can be improved in a similar fashion, e.g., by creating Au nanoparticles on the ND surface.

**Table 2:** Comparison of performances of sensors operated in the photo-activation mode at room temperature reported in the literature. Note that the listed responses are based on our present definition *S* = Δ*R*/*R*_0_ and some are different from the original values in the references.

material	gas	*C* (ppm)	*S* (%)	*t*_res_ (s)	*t*_rec_ (s)	light intensity (mW·cm^−2^)	ref.

CdS/ZnO NWs	NO_2_	1	337	40	230	0.68 (@ 468 nm)	[21]
Au-ZnO nanocomposites	NO	2	388	ca. 1000	—	— (@ 550 nm)	[20]
ZnO NWs SnO2 NWs SnO2/ZnO NWs	NO_2_	5	4 80 519	110 90 100	230 220 220	1.2 (@ 365 nm)	[17]
ZnO nanosheets Au/ZnO nanosheets	NO_2_	5	37 355	8 6	290 320	1.2 (@ 365 nm)	[18]
ZnO nanoline	NO_2_	20	108	ca. 600	ca. 300	25 (@ 365 nm)	[14]
TiO*_x_* ND	NO	10	31	91	184	0.3 (@ 310 nm)	this work

## Conclusion

In summary, single titanium oxide ND sensors are realized by AFM nanolithography and used for NO gas sensing. A Ti NW is generated first by AFM nanomachining and a titanium oxide ND is then produced in the NW by AFM nano-oxidation. With contact electrodes, a resistive ND gas sensor is created. For gas sensing at room temperature, light-assisted approaches, namely the UV-activation and the UV-recovery modes, are utilized. Two ND sensors have been fabricated. For the smaller sensor with a ND size of 80 nm, a response of 31%, a response time of 91 s, and a recovery time of 184 s have been achieved at 10 ppm NO in the UV-activation mode. For the larger sensor with a ND size of 120 nm, a response of 9%, a response time of 138 s, and a recovery time of 100 s have been achieved at 50 ppm NO in the UV-activation mode. The better performance of the smaller sensor can be attributed to the larger surface to volume ratio and smaller dimensions than the Debye length. The present work reveals the usefulness of single metal oxide NDs for gas sensing with reasonable performance that can be compared with metal oxide NW gas sensors.

## Supporting Information

Supporting Information features the current–voltage curves of sensors A and B before NO sensing, the resistances of sensors A and B before and after NO adsorption obtained from the current responses at a bias of 10 V as shown in the Figures, a finer time scale current response of sensor A at 10 V in the UV-recovery mode, the current response of sensor B at 5 V in the UV-activation mode, and the current response of sensor B at 10 V due to the injection of 500 ppm NO and subsequent high-pressure N_2_.

File 1 Additional experimental data.
