# Supplementary material for: NO gas sensing at room temperature using single titanium oxide nanodot sensors created by atomic force microscopy nanolithography
Source: Beilstein J Nanotechnol. 2016 Jul 20;7:1044–51. doi: 10.3762/bjnano.7.97 (PMC4979878; doi:10.3762/bjnano.7.97)
Supplement: File 1 — Additional experimental data. [file Beilstein_J_Nanotechnol-07-1044-s001.pdf]

Supporting Information

for

NO gas sensing at room temperature using single titanium oxide nanodot sensors created by atomic force microscopy nanolithography

Li-Yang Hong and Heh-Nan Lin*

Address: Department of Materials Science and Engineering, National Tsing Hua University, Hsinchu 30013, Taiwan

Email: Heh-Nan Lin*-hnlin@mx.nthu.edu.tw

*Corresponding author

Additional experimental data

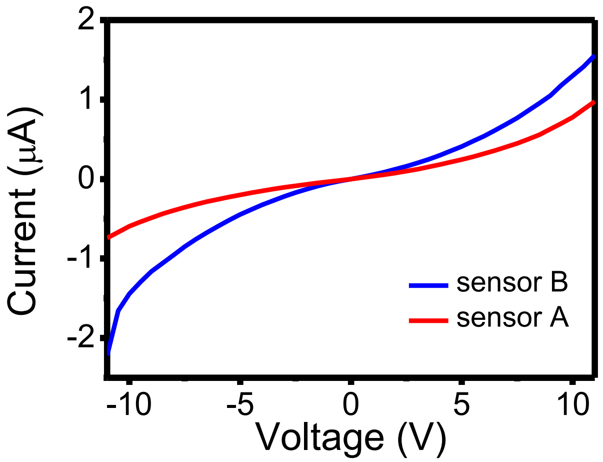

Figure S1: The current-voltage curves of sensors A and B before NO sensing.

Table S1: The resistances of sensors A and B before and after NO adsorption obtained from the current responses at a bias of 10 V as shown in the Figures.

sensor	C (ppm)	I_0 (μA)	I_g (μA)	R_0 (MΩ)	R_g (MΩ)	$\Delta R/R_0$ (%)
A	10	0.89	0.68	11.24	14.7	31
	15	0.89	0.63	11.24	15.87	41
	20	0.90	0.59	11.11	16.95	52
B	50	2	1.83	5	5.46	9
	100	2	1.72	5	5.81	16
	250	2	1.58	5	6.33	27
	500	2	1.36	5	7.35	47

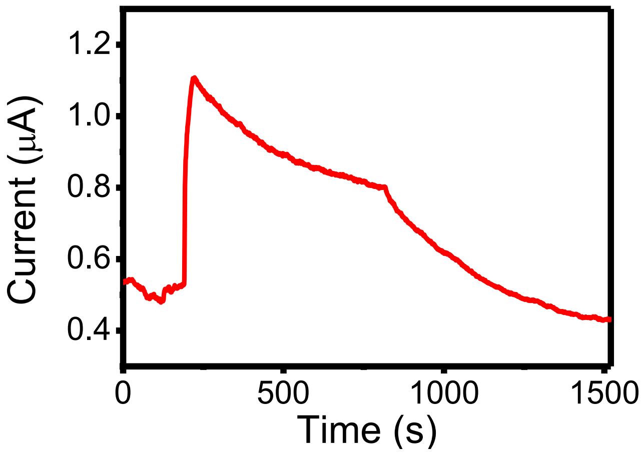

Figure S2: A finer time scale current response of sensor A at 10 V in the UV-recovery mode.

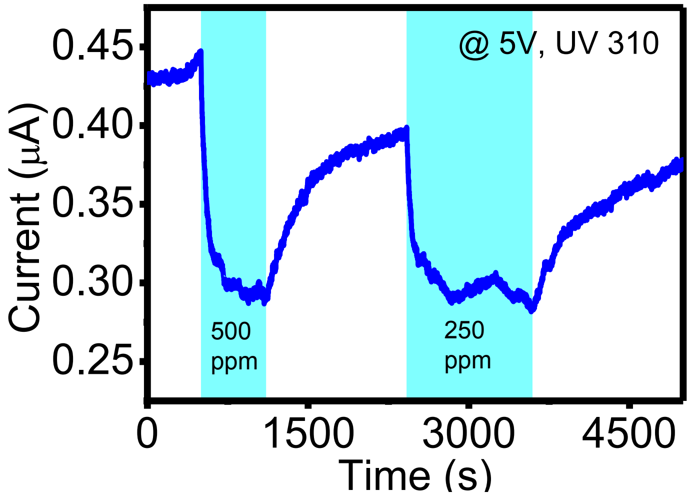

Figure S3: The current response of sensor B at 5 V in the UV-activation mode.

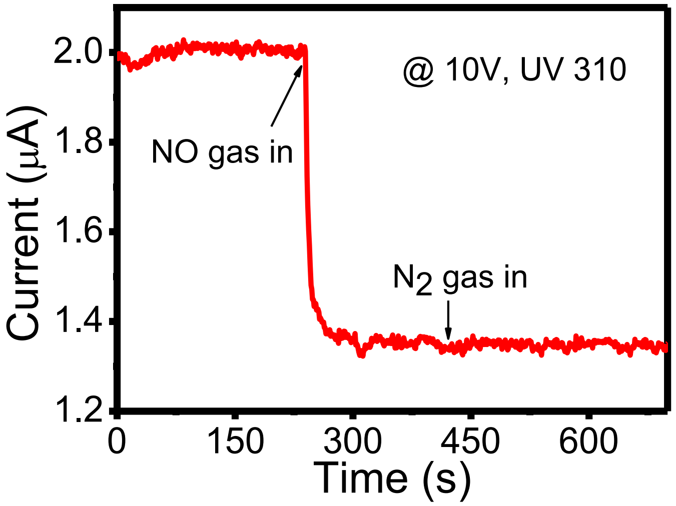

Figure S4: The current response of sensor B at 10 V due to the injection of 500 ppm NO and subsequent high-pressure N_2.
